# Low Sintering Temperature Effect on Crystal Structure and Dielectric Properties of Lead-Free Piezoelectric Bi_0.5_Na_0.5_TiO_3_-NaFeTiO_4_

**DOI:** 10.3390/ma17205087

**Published:** 2024-10-18

**Authors:** Luis G. Betancourt-Cantera, Yaneli Reséndiz-Trejo, Félix Sánchez-De Jesús, Claudia A. Cortés Escobedo, Ana M. Bolarín-Miró

**Affiliations:** 1 Área Académica de Ciencias de la Tierra y Materiales, Universidad Autónoma del Estado de Hidalgo, Mineral de la Reforma 42184, Mexico; betancourtcantera@gmail.com (L.G.B.-C.); re287151@uaeh.edu.mx (Y.R.-T.); fsanchez@uaeh.edu.mx (F.S.-D.J.); 2 Instituto Politécnico Nacional, Centro de Investigación e Innovación Tecnológica, Ciudad de México 02250, Mexico; claudia.alicia.cortes@gmail.com

**Keywords:** BNT, Bi_0.5_Na_0.5_TiO_3_, low temperature sintering, piezoelectric properties, BNT-NFT, dielectric properties

## Abstract

Bi_0.5_Na_0.5_TiO_3_ (BNT) emerges as a promising ferroelectric and piezoelectric lead-free candidate to substitute the contaminant Pb[Ti_x_Zr_1−x_]O_3_ (PZT). However, to obtain optimal ferroelectric and piezoelectric properties, BNT must be sintered at high temperatures. In this work, the reduction of sintering temperature by using iron added to BNT is demonstrated, without significant detriment to the dielectric properties. BNT-xFe with iron from x = 0 to 0.1 mol (∆x = 0.025) were synthesized using high-energy ball milling followed by sintering at 900 °C. XRD analysis confirmed the presence of rhombohedral BNT together with a new phase of NaFeTiO_4_ (NFT), which was also corroborated using optical and electronic microscopy. The relative permittivity, in the range of 400 to 500 across all the frequencies, demonstrated the stabilization effect of the iron in BNT. Additionally, the presence of iron elevates the transition from ferroelectric to paraelectric structure, increasing it from 330 °C in the iron-free sample to 370 °C in the sample with the maximum iron concentration (0.1 mol). The dielectric losses maintain constant values lower than 0.1. In this case, low dielectric loss values are ideal for ferroelectric and piezoelectric materials, as they ensure minimal energy dissipation. Likewise, the electrical conductivity maintains a semiconductor behavior across a range of 50 Hz to 1 × 10^6^ Hz, indicating the potential of these materials for applications at different frequencies. Additionally, the piezoelectric constant (d_33_) values decrease slightly when low concentrations of iron are added, maintaining values between 30 and 48 pC/N for BNT-0.025Fe and BNT-0.05Fe, respectively.

## 1. Introduction

Sodium bismuth titanate (BNT) is a ferroelectric material that has been broadly studied owing to its potential applications in different devices such as capacitors, piezoelectrics, semiconductors, and transducers [1,2,3]. Its significance stems from its notable spontaneous polarization (Ps) of approximately 35 µC/cm^2^ [4], combined with a substantial and stable dielectric constant of around 500 [5] across various frequency ranges with low energy dissipation. Additionally, one of the most compelling features of this material is its high ferroelectric Curie temperature of approximately 385 °C and its lead-free composition [6,7], a combination achieved by very few materials, making it suitable for use in biocompatible material applications and devices that operate at high temperatures [8,9].

BNT can be obtained through various methods, such as sol-gel, Pechini, and hydrothermal, among others [10,11,12,13]. However, the high production costs associated with these techniques make their scalability difficult. Therefore, a large amount of research work on BNT synthesis has focused on simpler methods, such as the solid-state reaction, including, in some cases, milling processes. Although this process ensures a homogeneous distribution of the oxide powders, the energy applied during milling is often insufficient to directly produce the BNT phase. To overcome this restriction, it is essential to heat treat the powder mixture at temperatures around 1100 °C. This additional step not only ensures proper crystallization and the development of the material functional properties but also helps to minimize porosity defects, which are crucial for maintaining optimal electrical performance [14]. Although high sintering temperatures are necessary, they are also counterproductive because Bi_2_O_3_ and Na_2_O have melting temperatures of 817 °C and 1132 °C, respectively [15,16], which can generate porosity and undesirable phases, leading to issues such as leakage current and increase in conductivity, which degrade the electrical properties and limit their applicability in the conception of devices. Additionally, operating at high temperatures to produce BNT results in high operational costs.

Given these challenges, some techniques have been proposed to reduce sintering temperature, preserving the dielectric and ferroelectric properties of the BNT. Among the most well-known techniques is spark plasma sintering (SPS), which provides significant advantages, including faster sintering rates and improved densification at reduced temperatures [17,18]. Similarly, nanometer-sized BNT powders obtained by chemical techniques hold great potential, offering benefits like the SPS method through their larger surface area and reactivity [19].

However, as mentioned earlier, the challenges in scaling the process limit its widespread applicability, highlighting the necessity for more efficient and feasible alternatives to advance the practical use of BNT. In this context, versatile alternatives such as high-energy milling can be an excellent method because it can help to create an amorphous phase that can act as an intermediate step before crystallization, reducing the energy needed to induce atomic rearrangement and form the crystalline BNT phase. On the other hand, doping BNT with specific ions has demonstrated promising results while reducing operational costs [20,21]. This effectiveness is because the introduction of dopants into the crystal structure of BNT can significantly reduce the sintering temperature by modifying grain boundaries, inducing lattice distortion, forming low-melting eutectic phases, and enhancing ionic diffusion [22,23,24].

Among the most used dopants for BNT are Mg, Al, Zr, and others [25,26,27]. Nevertheless, Fe^3+^ has shown particularly good results in lowering the sintering temperature of BNT because Fe^3+^ ions can enhance the atomic diffusivity during the sintering process. Additionally, Fe^3+^ ions can create oxygen vacancies, which can contribute to mass transport and grain boundary movement, leading to improved sintering results [28,29]. As a result, the material can achieve the desired densification and microstructure at a lower temperature. On the other hand, some studies have reported that doping BNT with Fe^3+^ enhances its ferroelectric properties by improving domain wall mobility, leading to enhanced electrical performance and stability, making Fe^3+^ a crucial dopant for optimizing the functional properties of BNT-based materials [30,31].

As mentioned earlier, in this study, we propose a simple method to obtain a lead-free ferroelectric material through high-energy milling and sintering at low temperatures. The addition of iron is expected to enhance this process by facilitating atomic diffusion. Additionally, the high-energy milling synthesis method is anticipated to induce atomic-level reactions that lead to the formation of an intermediate amorphous phase, thereby reducing the energy required for the formation of the BNT phase. Finally, this study includes an analysis to establish correlations between different concentrations of iron added and their impact on the crystalline structure, morphology, and dielectric and piezoelectric properties of BNT.

## 2. Materials and Methods

A specific stoichiometry was selected to compensate for the charges according to the composition Bi_0.5_Na_0.5_Ti_1−x_Fe_x_O_3−0.5x_ where x = 0.025, 0.05, 0.075, and 0.1 mol of Fe. High-purity oxide powders and carbonates Fe_2_O_3_, TiO_2_, Bi_2_O_3_, Na_2_CO_3_, and Ba_2_CO_3_, all from Sigma–Aldrich (St. Louis, MO, USA) with purities ranging from 99% to 99.8%, were used as precursors. Prior to the milling process, the precursors were dried at 150 °C for 1 h. Afterward, the precursors were mixed according to the stoichiometric reaction shown in the following equation:

(1) $$x{\mathrm{Fe}}_{2}O_{3}+0.5{\mathrm{Bi}}_{2}O_{3}+2\left(1-x\right){\mathrm{TiO}}_{2}+0.5{\mathrm{Na}}_{2}{\mathrm{CO}}_{3}\to \;2{\mathrm{Bi}}_{0.5}\mathrm{Na}0.5{\mathrm{Ti}}_{\left(1-x\right)}{\mathrm{Fe}}_{x}O_{3-0.5x}+0.5{\mathrm{CO}}_{2}$$

A total of 5 g of the powder mixture, along with steel balls of 12.7 mm with a ball-to-powder weight ratio of 10:1, was loaded into a steel vial of 50 cm^3^ in volume at room temperature in an air atmosphere. Subsequently, the mixture was high-energy ball milled by means of a shaker mixer mill (SPEX model 8000D, Metuchen, NJ, USA) at a speed of 1425 rpm for 5 h, with 30 min rest intervals after every 90 min of milling to prevent equipment overheating. After that, the milled powder was pressed into pellets using a hydraulic press (ENERPAC model PUJ1201B, Menomonee Falls, WI, USA), applying 1400 MPa for 15 min to obtain pellets of 10 mm in diameter. The pellets were sintered at 900 °C for 4 h using a tubular muffle furnace (Lindberg/Blue M model STF54459C, Riverside, MI, USA) in an air atmosphere.

The phases and crystal structures were determined by X-ray diffraction (XRD) using a diffractometer Equinox 2000 (INEL) (Artenay, Centre, France) with CoKα1 radiation (λ = 1.7018 Å). XRD patterns were measured in a 2ϴ range from 20° to 80°, followed by Rietveld refinement using free-software material analysis using diffraction (MAUD [32]) to study the evolution of the cell parameters, crystallite size, microstrain, and phase quantification as the iron concentration (x) was increased. The crystallographic powder diffraction (PDF) data was obtained from the Crystallography Open Database (COD) [33]. The pellets were painted with silver paste on both surfaces for electrical characterization. The loss tangent (tan δ), AC conductivity (σ_AC_), and relative permittivity were determined at room temperature. Additionally, the relative permittivity was evaluated within a temperature range from 25 °C to 400 °C using an LCR meter (HIOKI 3532-50, Nagano, Japan) within a frequency range of 50 Hz to 5 × 10^6^ Hz. To measure the piezoelectric constant (d_33_), the sintered pellets were subjected to poling under a direct current (DC) field of 4.5 kV/mm at room temperature. The d_33_ value was recorded 24 h after the poling process using a d_33_-meter model TYE2730 SINOCERA (NanChen Road, Shanghai, China). The surface morphology and microstructure of the samples were observed using both digital-optical (KEYENCE VHX-7000, Higashi-Yodogawa-ku, Osaka, Japan) and scanning electron microscopy (SEM, JEOL-100-CXII, Akishima, Tokyo, Japan), equipped with energy disperse spectroscopy (EDS) analysis to perform elemental analysis.

## 3. Results and Discussion

### 3.1. Crystal Structure

**Figure 1:**
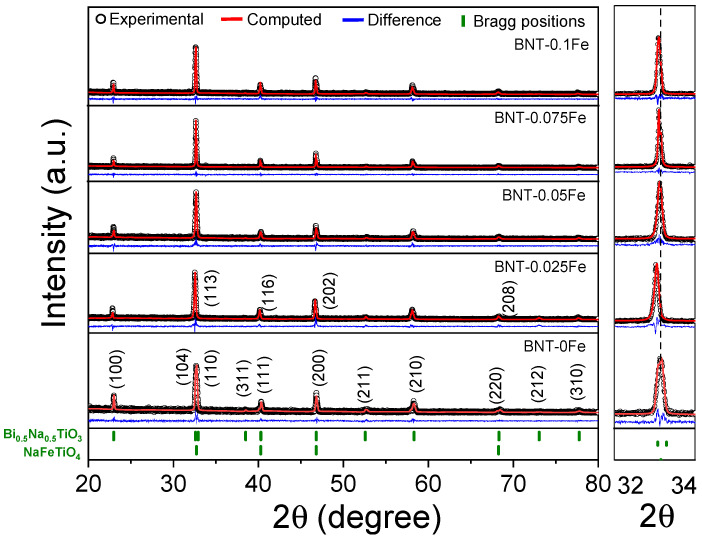
XRD patterns of pellets of mixtures of Bi_2_O_3_, Fe_2_O_3_, Na_2_CO_3_, and TiO_2_ milled for 5 h and sintered at 900 °C for obtaining Bi_0.5_Na_0.5_Ti_1−x_FexO_3−0.5x_ (0 ≤ x ≤ 0.1, Δx = 0.025).

Figure 1 shows the X-ray diffraction (XRD) patterns of sintered BNT samples with different iron concentrations (0.025, 0.05, 0.075, and 0.1 mol) obtained through high-energy ball milling and sintered at a low temperature of 900 °C. The analysis of the XRD profile for BNT-0Fe indicates the presence of a single-phase compound corresponding to the Bi_0.5_Na_0.5_TiO_3_ (BNT), which crystallizes in a rhombohedral structure with space group *R3c* (ICSD # 2103295). This structure is distinguished by a slight tilting of the TiO_6_ octahedra, leading to distinct structural distortions.

In contrast, when iron is added at a concentration of 0.025 mol into the BNT, a new phase, identified as NaFeTiO_4_ (ICSD # 2002628, NFT) and characterized by an orthorhombic structure and space group *P1*, is detected. Additionally, it is observed that as the iron concentration increases, the diffraction peaks associated with the NaFeTiO_4_ phase become more intense, indicating an increment in the wt. % of this phase. However, it is not easily distinguishable in the diffraction profiles, as its characteristic peaks overlap with those of the BNT phase due to similar diffraction angles. These results are compelling, indicating that Fe^3+^ ions partially substitute for Ti^4+^ within the BNT crystal lattice. However, instead of fully integrating, the iron is segregated, leading to the formation of a distinct secondary phase. Based on the obtained results, reaction (1) was not completed due to the diffusion process during sintering; therefore, the reaction shown in Equation (2) is proposed instead:

(2) $$(2+x){\mathrm{Fe}}_{2}O_{3}+0.5{\mathrm{Bi}}_{2}O_{3}+\left(1-2x\right){\mathrm{TiO}}_{2}+{\mathrm{Na}}_{2}{\mathrm{CO}}_{3}\to \;2{\mathrm{Bi}}_{0.5}{\mathrm{Na}}_{0.5}{\mathrm{Ti}}_{\left(1-x\right)}{\mathrm{Fe}}_{x}O_{3-0.5x}+\mathrm{Na}\mathrm{Fe}{\mathrm{TiO}}_{4}+0.5{\mathrm{CO}}_{2}$$

In this context, concerning the main phase, the BNT consistently maintains its rhombohedral *R3c* structure across all compositions. This suggests that the partial substitution of Fe^3+^ does not significantly alter the overall symmetry of the crystal lattice, ruling out the possibility of phase transformations. Nevertheless, the structural defects induced by the Fe^3+^ are sufficient for the diffraction peaks to exhibit a perceptible shift as the iron concentration increases, revealing a displacement toward low angles with respect to 2θ. The observed behavior is attributed to the substitution of Ti^4+^ by Fe^3+^ ions, resulting in an expansion of the unit cell due to the larger ionic radius of Fe^3+^ (0.64 Å) compared to Ti^4+^ (60 Å) along with the ionic charge, a phenomenon explained by Bragg’s law as shown in Equation (3):

(3) nλ=2dsin θ

where n is the order of reflection, λ is the wavelength of the X-rays, d is the interplanar spacing, and θ is the diffraction angle. Thus, as the lattice expands, the interplanar spacing d increases, causing a decrease in the diffraction angle 2θ to maintain the Bragg condition.

Furthermore, Table 1 presents the Rietveld refinement parameters obtained from the X-ray diffraction profiles corresponding to Figure 1. As was expected from the XRD patterns, the iron-free sample exhibits a 100 wt. % of the Bi_0.5_Na_0.5_TiO_3_ phase without the presence of secondary phases. However, after adding iron at a concentration of 0.025 mol, the new phase corresponding to NaFeTiO_4_ is quantified as 5.67 wt. %, reaching a maximum of 37.65 wt. % in the sample with the highest iron concentration, BNT-0.1Fe. It confirms the formation of a BNT-NFT composite by a simple high-energy milling followed by sintering at 900 °C. Concerning the lattice parameters of the BNT phase, a gradual increase was observed, like the progressive shift in diffraction peaks as the iron concentration increases, which is attributed to the partial substitution of Ti^4+^ by Fe^3+^, as previously discussed. The low χ^2^ and R_wp_ values suggest minimal discrepancies, ensuring that the refinement results are robust and consistent with the expected structural characteristics.

### 3.2. Morphological Analysis

**Figure 2:**
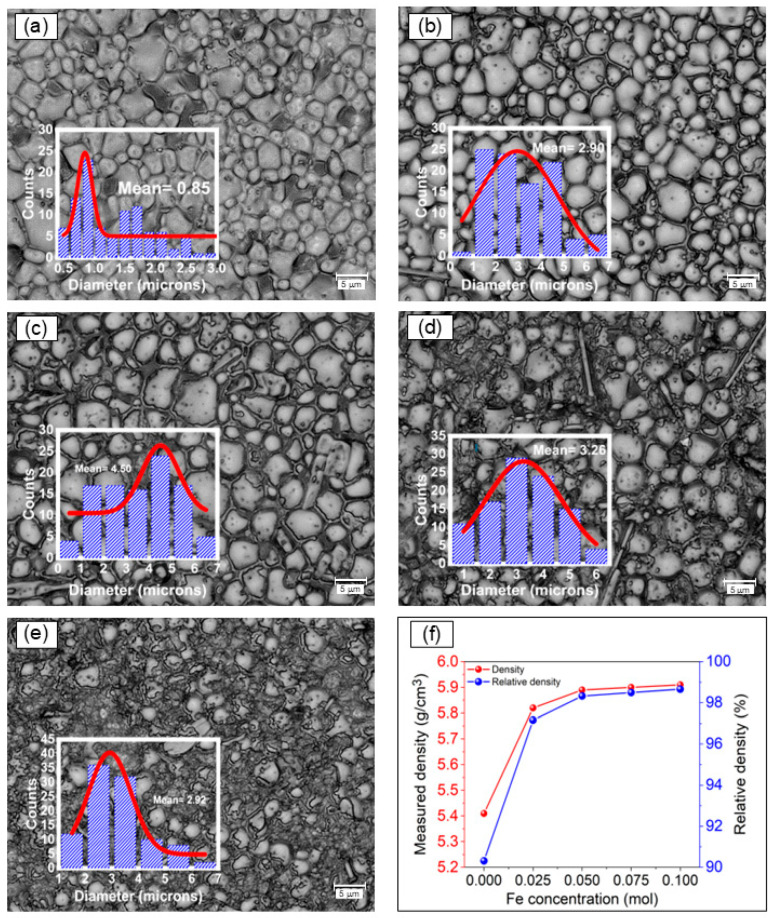
Digital-optical micrographs of pellets obtained from mixtures of Bi_2_O_3_, Fe_2_O_3_, Na_2_CO_3_, and TiO_2_ milled for 5 h and sintered at 900 °C for obtaining Bi_0.5_Na_0.5_Ti_1−x_Fe_x_O_3−0.5x_ with x of: (**a**) x = 0, (**b**) x = 0.025, (**c**) x = 0.05, (**d**) x = 0.075 and (**e**) x = 0.1, (**f**) Densities of the BNT-xFe samples.

Figure 2 shows optic-digital micrographs of the BNT-NFT sintered pellets with different amounts of iron. These digital micrographs allow visualization of the phase distribution and have a much larger depth of field than conventional optical microscopes.

As can be observed from the surface morphologies of the samples, all the studied compositions are formed by homogeneous quasi-equiaxial grains, although it is important to note that the iron-free sample exhibits small pores, likely due to incomplete densification, which can be attributed to the low sintering temperature of 900 °C. Notably, two significant effects are observed following iron addition. First, a notable outcome is the gradual increase in the intergranular phase correlated with the iron-rich phase, NFT, attributed to the higher atomic diffusion rates induced by the partial incorporation of Fe^3+^, which lowers the energy barriers for grain boundary movement. Another important observation is the substantial reduction in porosity defects with respect to the iron-free sample (Figure 2a). This reduction directly contributes to increasing the material’s density, as is evidenced in the micrographs presented in Figure 2, which includes the quantified density of the sintered pellets as inset. In this case, it reflects a more efficient sintering process improved by the partial incorporation of iron into the BNT lattice, contributing to the overall mechanical strength and the electrical performance of the material.

The improvement in the sintering process is further supported by the data, as shown in the inset and graph in Figure 2. It is clearly observed that both grain size and density increase with the addition of iron, as mentioned earlier. However, starting from the BNT-0.075Fe sample, it cannot be measured accurately with this technique due to a different phase morphology overlapping the equiaxial grains. In this scenario, the overlapping phases correspond to the NaFeTiO_4_ phase, which arises when iron is added. This phase grows from the grain boundaries and becomes more pronounced as the iron content increases, as shown in Figure 2.

**Figure 3:**
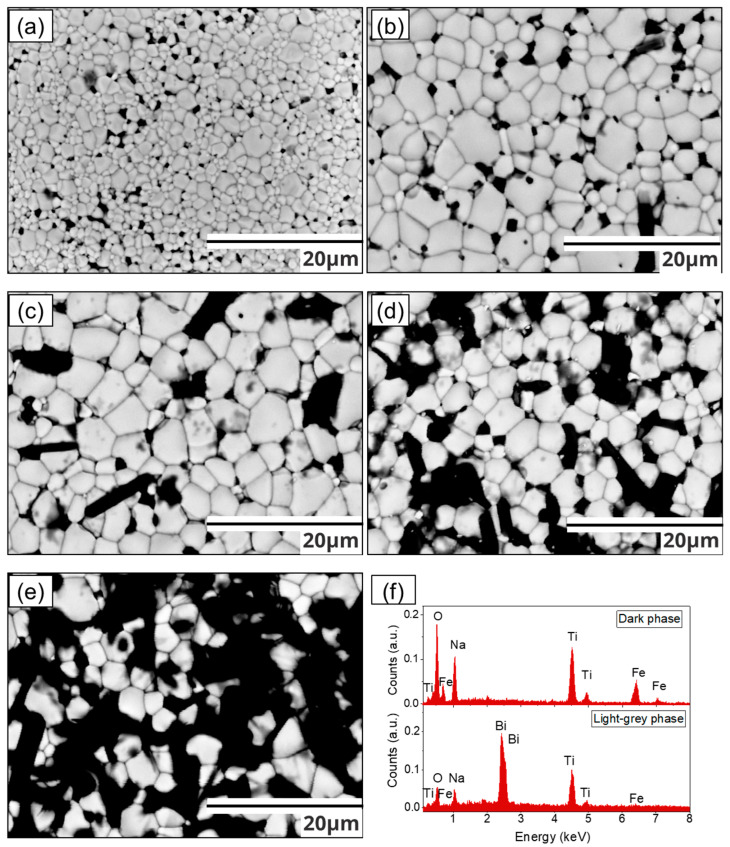
Scanning electronic micrographs of pellets obtained from mixtures of Bi_2_O_3_, Fe_2_O_3_, Na_2_CO_3_, and TiO_2_ milled for 5 h and sintered at 900 °C for obtaining Bi_0.5_Na_0.5_Ti_1−x_Fe_x_O_3−0.5x_ with x of: (**a**) x = 0, (**b**) x = 0.025, (**c**) x = 0.05, (**d**) x = 0.075, (**e**) x = 0.1 and (**f**) EDS analysis of two distinct regions or phases.

**Figure 4:**
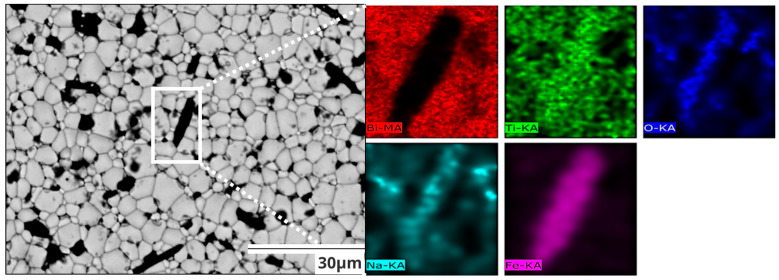
Scanning electron micrograph at 2000× showing a surface of BNT sample with 0.05 mol Fe and the associated energy dispersive spectroscopy (EDS) elemental analysis mapping of the selected area of the micrograph (square) for bismuth (Bi—red), titanium (Ti—green), oxygen (O—dark blue), sodium (Na—blue) and iron (Fe—purple).

In order to corroborate the presence of precipitates, secondary phases, or simply change in the morphology, scanning electronic micrographs of the surface of the pellets obtained with retro-dispersed electrons are presented in Figure 3. In agreement with the optical-digital micrographs shown in Figure 2, all compositions are characterized by poly-equiaxial grains, whose growth is facilitated by the substitution of Fe^3+^ by Ti^4+^ in the BNT crystal structure. Additionally, the presence of a polygonal black phase is detected (NFT), which emerges from the grain boundaries and increases as the iron content increases, in agreement with the previous observation in the optical-digital micrographs shown in Figure 2 in contrast to the bright phase corresponding to the heavier BNT phase. Figure 3f and Figure 4 include an energy dispersive spectroscopy (EDS) analysis of the different grains observed in the sintered pellets, which provides some insight into the composition of the studied materials. In this context, the elemental analysis (mapping) shown in Figure 4 provides crucial evidence to identify the presence of the NaFeTiO_4_ phase (black grains), which would otherwise be undetectable by XRD due to the overlap of its characteristic diffraction peaks with those of the BNT phase, revealing iron segregation, which contributes to its formation. Meanwhile, the gray phase corresponds to the BNT phase without a significant amount of iron atoms.

### 3.3. Dielectric Properties

**Figure 5:**
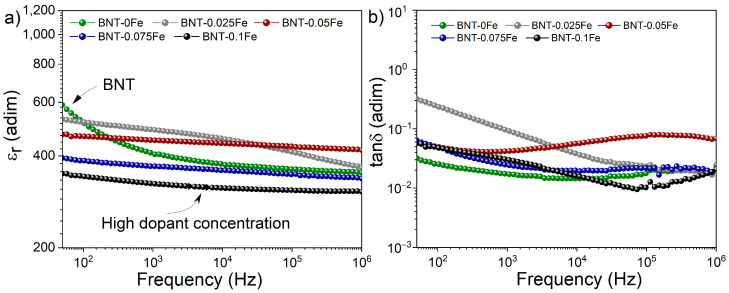
Frequency-dependence of (**a**) relative permittivity (ε_r_) and (**b**) loss tangent (tan δ) at room temperature of pellets obtained from mixtures of Bi_2_O_3_, Fe_2_O_3_, Na_2_CO_3_, and TiO_2_ milled for 5 h and sintered at 900 °C for obtaining Bi_0.5_Na_0.5_Ti_1−x_FexO_3−0.5x_ with x from 0 to 0.1 with ∆x = 0.025.

The effect of iron on the relative permittivity (ε_r_) and tan δ versus the frequency of BNT at room temperature is shown in Figure 5a. As can be seen, the iron-free sample reveals an initial ε_r_ of 600; however, as the frequency increases, this value drops abruptly, remaining almost horizontal around 450 at frequencies above 10⁴ Hz. This phenomenon is associated with the interfacial polarization mechanism, where the primary contributors to polarization at low frequencies are volumetric and surface defects, such as porosity observed in Figure 2. These defects can act as pinning centers for electric charges, thereby limiting their mobility and contributing to the overall ε_r_. In this context, although the ε_r_ value might seem relatively high, it does not reflect the inherent polarization of the BNT material at low frequencies.

In contrast, the samples containing NFT exhibit remarkably steady ε_r_ behavior across the entire frequency range studied, with values between 300 and 400. This steadiness suggests a reduction in porosity-related defects in the pellets with iron, as revealed in the micrographs in Figure 2 and Figure 3. Therefore, it can be inferred that the primary polarization mechanisms contributing to ε_r_ are ionic in nature. Additionally, a gradual decrease in ε_r_ is observed with increasing iron concentration due to the partial incorporation of iron into the BNT structure, which generates defects such as oxygen vacancies that deteriorate the dielectric properties of the material. Furthermore, the presence and gradual increase of NaFeTiO_4_ can disrupt the continuity of the BNT phase, creating interfaces that hinder the movement of electric dipoles, limiting the material’s ability to polarize efficiently under an applied electric field, thereby contributing to the observed reduction in the ε_r_.

Figure 5b illustrates the energy dissipation or dielectric losses in BNT samples with iron (x = 0, 0.025, 0.05, 0.075, and 0.1 mol) across a range of 50 Hz to 1 × 10^6^ Hz of frequency, expressed as tan δ. As observed, the results reveal fluctuations in tan δ values, particularly at higher iron concentrations, which can further explain the dielectric losses at high frequencies, revealing the presence of dielectric relaxations attributed to the lag between the applied electric field and the alignment of dipoles within the material. Hence, at high frequencies, the electric field changes direction more rapidly, making it difficult for the dipoles to realign quickly enough to keep up with the oscillating field. Additionally, these relaxations may also be linked to the presence of defects, such as oxygen vacancies introduced by iron, which can create localized states within the material that contribute to the observed relaxation behavior at higher frequencies. Despite this effect, the low tan δ values, ranging from 0.05 to 0.1 in the BNT-NFT samples, are not much higher than those observed in iron-free BNT. This indicates that the synthesized materials are efficient in terms of electrical energy storage, making them promising candidates for ferroelectrics and piezoelectric applications.

**Figure 6:**
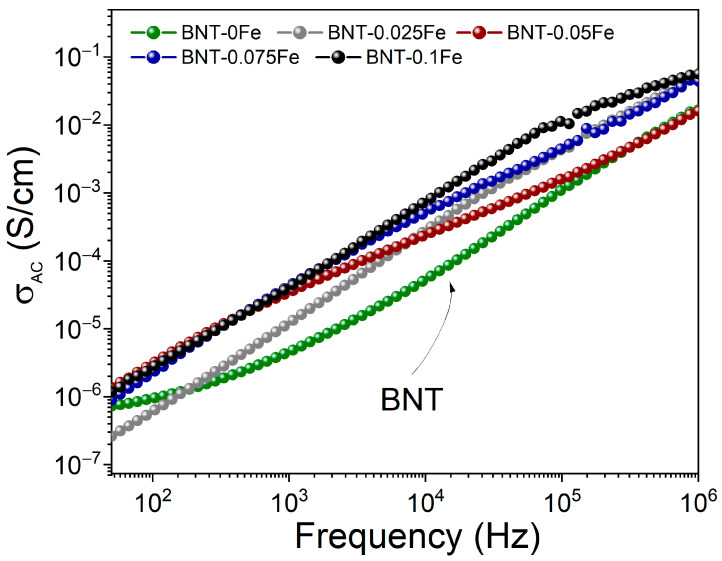
Conductivity vs. frequency of the BNT-xFe samples with different Fe concentrations with x from 0 to 0.1 with ∆x = 0.025.

Figure 6 shows the electrical conductivity behavior versus the frequency for the BNT samples with iron at different concentrations (0, 0.025, 0.05, 0.075, and 0.1 mol). As observed, the BNT-0Fe sample exhibits a conductivity of 10^−7^ S/cm at 50 Hz, which gradually increases as a function of frequency, reaching 10^−2^ S/cm at 10^6^ Hz. On the other hand, adding iron at a 0.025 mol concentration to the stoichiometry slightly reduces the conductivity values at 50 Hz compared to the iron-free BNT. Although higher conductivity at low frequencies would be anticipated due to charge compensation mechanisms like oxygen vacancies generated by partial iron substitution and the presence of the NFT phase, the observed reduction can be attributed to dislocations in the BNT crystal structure, which hinder the mobility of charge carriers and disrupt conduction pathways at low frequencies.

Subsequently, the BNT-0.05Fe sample exhibits increased conductivity due to the higher concentration of oxygen vacancies and the presence of the secondary phase NFT, both of which contribute to elevated conductivity values. However, at a frequency of 10^3^ Hz, this sample displays a unique behavior compared to the others as its conductivity gradually decreases, eventually reaching values similar to those of the undoped BNT sample at higher frequencies. This behavior is linked to the interfaces between the two phases at these particular proportions, as well as the structural defects created, which together can act as barriers for charge carriers, disrupting their movement as the frequency increases. Consequently, conductivity decreases. For the BNT samples with high concentrations of iron (0.075 and 0.1 mol), it is observed that the conductivity is significantly higher and more stable across the studied frequency range compared to other concentrations. This behavior can be attributed to the increased number of oxygen vacancies generated by the high iron concentration, which facilitates increased mobility of charge carriers

Finally, it should be noted that the conductivity values fall within the semiconductor range, and this behavior is consistently maintained from 50 Hz to 1 × 10^6^ Hz. This suggests that the material can operate effectively across a wide range of frequencies, making it suitable for various electronic applications.

### 3.4. Temperature-Dependent Dielectric Properties

**Figure 7:**
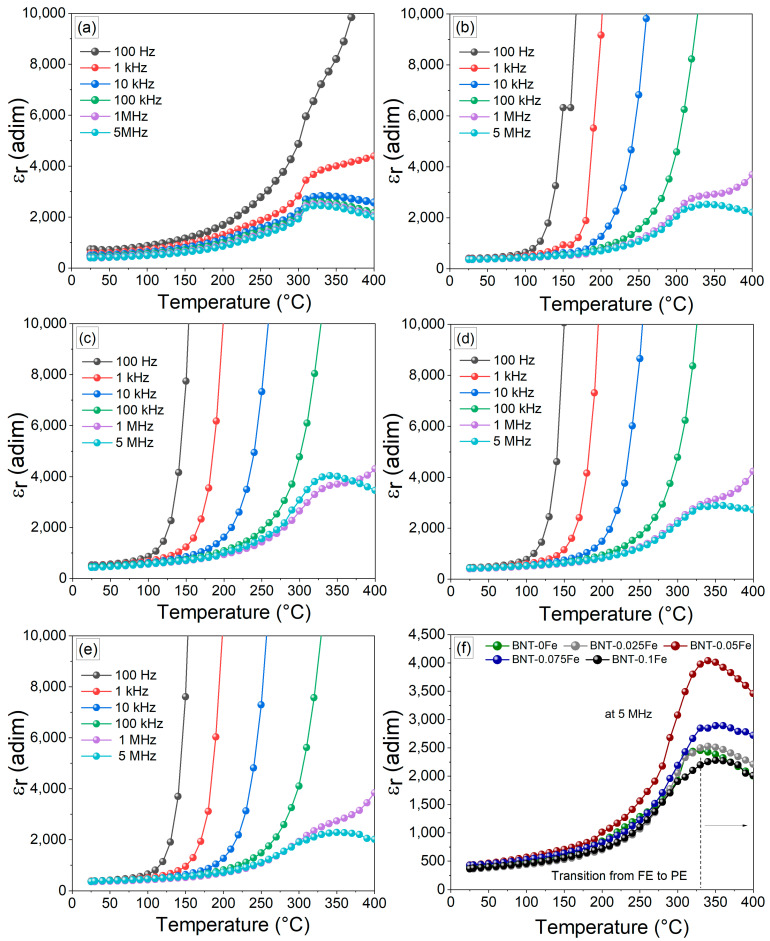
Temperature dependence of relative permittivity (ε_r_) at different frequencies, from 100 Hz to 5 MHz, of pellets obtained from mixtures of Bi_2_O_3_, Fe_2_O_3_, Na_2_CO_3_, and TiO_2_ milled for 5 h and sintered at 900 °C for obtaining Bi_0.5_Na_0.5_Ti_1−x_Fe_x_O_3−0.5x_, with x values as follows: (**a**) x = 0, (**b**) x = 0.025, (**c**) x = 0.05, (**d**) x = 0.075, and (**e**) x = 0.01. (**f**) Transition temperature from ferroelectric (FE) to paraelectric (PE) of different iron compositions at 5 MHz.

The temperature dependence of the relative dielectric permittivity (ε_r_) and the loss tangent (tan δ) for BNT-xFe ceramics at various frequencies (100 Hz, 1 kHz, 10 kHz, 100 kHz, 1 MHz, and 5 MHz) is illustrated in Figure 7. As can be appreciated, the iron-free sample exhibits a significant increase in the ε_r_ as the temperature rises. This behavior is typical of ferroelectrics and can be attributed to the enhanced alignment of electric dipoles facilitated by thermal energy [34]. Consequently, as the temperature continues to rise, the material undergoes a phase transition from ferroelectric to paraelectric at 330 °C, reaching a maximum ε_r_ of 2400 at 5 MHz. For samples with low concentrations of iron (0.025 and 0.05 mol), a notable increase in ε_r_ values is observed, reaching up to 4048 in the BNT-0.05Fe sample. This effect may be related to the ability of iron, at low concentrations, to facilitate the alignment of electric dipoles under the applied field, as mentioned previously. Additionally, there is a gradual increase in the transition temperature from ferroelectric to paraelectric, likely due to the rise in structural defects induced by iron in the BNT phase. These defects can stabilize the ferroelectric phase by creating a more favorable energetic configuration for dipolar alignment, requiring higher thermal energy to induce the transition from the ferroelectric to the paraelectric phase. This trend continues in the subsequent samples, BNT-0.075Fe and BNT-0.1Fe, reaching up to 370 °C in the latter. Nevertheless, the results show that exceeding the iron content of 0.05 mol is counterproductive for the dielectric properties, as it leads to a reduction in permittivity values. Beyond this threshold, the ε_r_ decreases to values below those observed in the iron-free sample. It is important to note that the presence of iron at low concentrations leads to its improvement, thereby expanding the potential applications of the BNT-NFT system processed at low temperatures.

**Figure 8:**
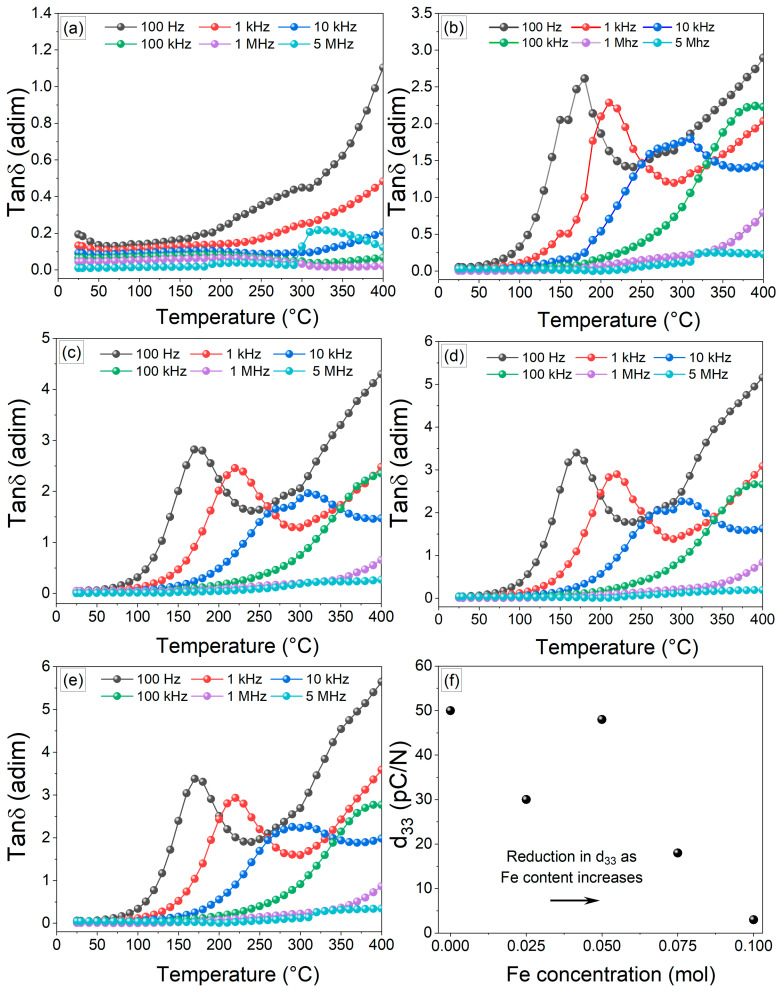
Temperature dependence of dielectric, tanδ, at different frequencies, from 100 Hz to 5 MHz, of pellets obtained from mixtures of Bi_2_O_3_, Fe_2_O_3_, Na_2_CO_3_, and TiO_2_ milled for 5 h and sintered at 900 °C for obtaining Bi_0.5_Na_0.5_Ti_1−x_FexO_3−0.5x_, with x values as follow: (**a**) x = 0, (**b**) x = 0.025, (**c**) x = 0.05, (**d**) x= 0.075 (**e**) x = 0.01, and (**f**) piezoelectric constant (d_33_) of Bi_0.5_Na_0.5_Ti_1−x_Fe_x_O_3−0.5x_ samples with different iron concentrations (x = 0, 0.025, 0.05, 0.075, and 0.1 mol).

Figure 8 illustrates the tanδ measured at various temperatures across specific frequencies, ranging from 100 Hz to 5 MHz. In the BNT-0Fe sample, the tanδ values increase with rising temperature at lower frequencies, such as 100 Hz and 1 kHz. This phenomenon occurs because the dipoles have enough time to respond to fluctuations in the electric field, aided by the thermal energy, which facilitates enhanced polarization. However, this also causes increased dielectric losses. Conversely, at higher frequencies, this effect is significantly diminished. As the frequency increases, the time available for the dipoles to align with the electric field decreases, preventing them from keeping pace with the rapid changes in the field.

Consequently, this reduces energy dissipation, allowing for more stable values within the examined temperature range. Conversely, samples with iron content show a significant increase in dielectric losses due to the presence of charge carriers like oxygen vacancies. Additionally, dielectric relaxations are noticeable, manifested as abrupt peaks. This behavior can be attributed to the partial incorporation of iron into the crystal structure of BNT, which can promote enhanced resonance in the dipoles, allowing it to align more effectively with the applied electric field at specific temperatures, leading to a sharp increase in dielectric losses. Finally, in Figure 8f, the piezoelectric constant of the BNT-xFe samples is shown. The undoped BNT exhibited a piezoelectric constant of 50 pC/N. Similarly, samples with low iron concentrations displayed comparable piezoelectric values, benefiting from improved density that enhances mechanical properties, making them suitable for advanced applications. In contrast, higher iron concentrations led to a decline in d_33_ values, likely due to defects introduced in the crystal lattice and NFT phase.

## 4. Conclusions

BNT milled with different amounts of iron, ranging from 0 to 0.1 mol of Fe, was subjected to high-energy ball milling followed by sintering at temperatures as low as 900 °C. XRD patterns demonstrate that the resultant ceramics display a composite formed by a mixture of rhombohedral and orthorhombic phases, corresponding to the presence of Bi_0.5_Na_0.5_TiO_3_ (BNT) and NaFeTiO_4_ (NFT) compounds, respectively. The surface morphology, observed through optical and electron microscopy, validated the presence of a uniform distribution of grains composed of a mixture of both phases, a reduction in porosity, and an increase in grain size with the increase in iron content, corroborating the positive effect of the iron in the density of sintered pellets. Furthermore, the study of relative permittivity versus temperature confirmed an increase in the transition temperature from ferroelectric to paraelectric order with increasing iron content, maintaining low dielectric loss values. Finally, d_33_ piezoelectric constant measurements show that at low iron concentrations (0.025 and 0.05 mol), it is possible to maintain values close to the undoped sample (~50 pC/N). However, as the iron content increases to 0.075 and 0.1 mol, a significant decrease in d_33_ values is observed, dropping to 15 and 3 pC/N, respectively. This reduction is likely due to the introduction of defects or structural changes caused by the higher iron content, which adversely impacts the piezoelectric properties of the material.

## Figures and Tables

**Table 1:** Rietveld refinement parameters obtained from the X-ray diffraction patterns of the pellets of mixtures of Bi_2_O_3_, Fe_2_O_3_, Na_2_CO_3_, and TiO_2_ milled for 5 h and sintered at 900 °C for obtaining Bi_0.5_Na_0.5_Ti_1−x_Fe_x_O_3−0.5x_ (0 ≤ x ≤ 0.1, Δx = 0.025).

Sample Reference	Phase/Space Group	Phase (wt. %)	Crystallite Size (nm)	Microstrain	Cell Parameters (Å)			Goodness of Fit	
					a	b	c	R_wp_	χ^2^
BTO-0Fe	BNT/*R3c*	100	137.30 ± 15.59	9.1041 × 10^−4^ ± 8.0735 × 10^−5^	5.4881 ± 3.3856 × 10^−5^	5.4881 ± 3.3856 × 10^−5^	13.5344 ± 1.3640 × 10^−5^	1.11	26.39
BTO-0.025Fe	BNT/*R3c*	94.32	289.47 ± 38.42	9.3530 × 10^−4^ ± 4.4587 × 10^−5^	5.4948 ± 3.1785 × 10^−5^	5.4948 ± 3.1785 × 10^−5^	13.5579 ± 1.4802 × 10^−5^	1.17	22.78
	NFT/*P1*	5.67	43.13 ± 8.15	2.6436 × 10^−5^ ± 1.3672 × 10^−6^	3.9894 ± 3.1785 × 10−^4^	9.5146 ± 1.5705 × 10^−5^	11.0239 ± 4.3150 × 10^−5^		
BTO-0.05Fe	BNT/*R3c*	83.11	225.65 ± 23.97	10.6714 × 10^−4^ ± 1.0417 × 10^−5^	5.4902 ± 1.1840 × 10^−5^	5.4902 ± 1.1670 × 10^−5^	13.5478 ± 4.8532 × 10^−5^	1.14	27.61
	NFT/*P1*	16.89	967.93 ± 20.44	1.0854 × 10^−4^ ± 3.6401 × 10^−5^	4.5834 ± 1.7094 × 10^−5^	9.5721 ± 8.5252 × 10^−5^	11.8484 ± 1.5129 × 10^−5^		
BTO-0.075Fe	BNT/*R3c*	72.24	361.89 ± 21.94	11.5351 × 10^−4^ ± 2.8269 × 10^−5^	5.4915 ± 1.7874 × 10^−5^	5.4915 ± 1.7874 × 10^−5^	13.5475 ± 9.3411 × 10^−4^	1.09	25.53
	NFT/*P1*	27.76	99.28 ± 8.54	5.0834 × 10^−4^ ± 4.5800 × 10^−5^	4.6395 ± 1.9070 × 10^−5^	9.5865 ± 8.5251 × 10^−5^	11.9140 ± 5.1200 × 10^−5^		
BTO-0.1Fe	BNT/*R3c*	62.35	355.31 ± 17.20	11.7322 × 10^−4^ ± 1.7723 × 10^−5^	5.4924 ± 1.4701 × 10^−5^	5.4924 ± 1.4701 × 10^−5^	13.5530 ± 1.4701 × 10^−5^	1.18	25.44
	NFT/*P1*	37.65	164.31 ± 19.23	7.1245 × 10^−4^ ± 9.2101 × 10^−5^	4.6585 ± 2.7018 × 10^−5^	9.5898 ± 3.0401 × 10^−5^	11.9832 ± 3.9850 × 10^−5^		

BNT: Bi_0.5_Na_0.5_TiO_3_ and NFT: NaFeTiO_4._

## Data Availability

The raw and processed data required to reproduce these findings are available to download from https://data.mendeley.com/preview/gmt8w495pm?a=53519b5a-731b-4f7e-ae6b-6dfdc5d9d2ca (accessed on 15 October 2024).
